# Bortezomib Prevents Acute Doxorubicin Ovarian Insult and Follicle Demise, Improving the Fertility Window and Pup Birth Weight in Mice

**DOI:** 10.1371/journal.pone.0108174

**Published:** 2014-09-24

**Authors:** Elon C. Roti Roti, Ashley K. Ringelstetter, Jenna Kropp, David H. Abbott, Sana M. Salih

**Affiliations:** 1 Department of Obstetrics & Gynecology, University of Wisconsin-Madison, Madison, Wisconsin, United States of America; 2 Department of Medicine, University of Wisconsin-Madison, Madison, Wisconsin, United States of America; 3 Wisconsin National Primate Research Center, University of Wisconsin-Madison, Madison, Wisconsin, United States of America; 4 University of Wisconsin-Madison, Department of Animal Sciences, Madison, Wisconsin, United States of America; Zhejiang University School of Medicine, China

## Abstract

Increasing numbers of female patients survive cancer, but succumb to primary ovarian insufficiency after chemotherapy. We tested the hypothesis that Bortezomib (Bort) protects ovaries from doxorubicin (DXR) chemotherapy by treating female mice with Bort 1 hour prior to DXR. By preventing DXR accumulation in the ovary, Bort attenuated DXR-induced DNA damage in all ovarian cell types, subsequent γH2AFX phosphorylation, and resulting apoptosis in preantral follicles. Bort pretreatment extended the number of litters per mouse, improved litter size and increased pup weight following DXR treatment, thus increasing the duration of post-chemotherapy fertility and improving pup health. As a promising prophylactic ovoprotective agent, Bort does not interfere with cancer treatment, and is currently used as a chemotherapy adjuvant. Bort-based chemoprotection may preserve ovarian function in a non-invasive manner that avoids surgical ovarian preservation, thus diminishing the health complications of premature menopause following cancer treatment.

## Introduction

Improvements in cancer detection and treatment have dramatically increased post-cancer life expectancy. Ironically, cancer survivors become lifelong patients due to detrimental chemotherapy (chemo) effects on otherwise healthy organs [1]. Primary ovarian insufficiency (POI) caused by chemo insult is an imminent women's health concern, affecting over 8% of female childhood cancer survivors (currently 1 in 400 adults) and 40% of breast cancer survivors [1]–[4]. Premature menopause resulting from POI causes infertility and increases a woman's risk of complications from estrogen deficiency, including osteoporosis, mental health disorders, and cardiovascular disease. Ovarian protection and fertility preservation are particularly limited for children and adolescent girls for whom the only available prophylactic requires surgically removing and freezing ovarian tissue for future re-transplantation [5]. While re-transplanted tissue provides transient return of menstrual hormone cycles, it is an expensive, invasive, experimental procedure that risks reintroducing the original cancer back into the patient at the time of re-transplantation [2], [5]. Oocyte and embryo cryopreservation are established procedures for women, but require expensive treatments that delay cancer therapy and do not preserve estrogen production [6]. Promising animal and *in vitro* studies have identified potential fertoprotective agents including FTY720, dexrazoxane, AS101, sphingosine-1-phosphate, imatinib, tamoxifen, and goserelin, [7]–[13], but none have yet been implemented in clinical practice. A drug-based ovarian shield (ovoprotective agent) administered as a prophylactic has potential to *prevent* ovarian chemo toxicity, and maintain reproductive health, including endocrine function and fertility, for all pre-menopausal cancer survivors regardless of cancer type.

Doxorubicin (DXR) is an anthracyline used to treat ∼50% of all cancers, including breast, bladder, and thyroid cancer (11% of adolescent cancer), leukemia (31% of pediatric, 12% of adolescent), lymphoma (10% of pediatric, 23% of adolescent), neuroblastoma (7% of pediatric), and Wilm's tumors (5% of pediatric) [14]–[16]. The use of DXR results in higher survival rates than other therapies, creating demand for a prophylactic ovarian shield to prevent DXR-induced POI. While DXR causes cell death via two distinct mechanisms, inducing oxidative stress and double-strand DNA breaks [17]–[19], the primary mode of DXR damage to the ovary involves formation of Topo-II-dependent dsDNA breaks [10] following DXR transport into the cell nucleus and DNA intercalation.

DXR causes follicular apoptosis in mouse ovaries by 12 hours (hrs) post-injection, with a return to 50% pre-DXR ovulation rate at 1 month post-DXR and long-term follicle depletion [20]–[22]. To generate an *in vivo* model for testing putative ovoprotective agents, we previously characterized acute DXR accumulation in the mouse ovary, subsequent DNA damage, and apoptosis [23]. DXR accumulates first in stromal cells, then progressively shifts radially inward, penetrating follicles. Given this temporal pattern of ovarian insult, DXR-induced dsDNA breaks not surprisingly occur first in stromal cells followed by granulosa cells that nurture oocytes. Ovoprotective agents must therefore protect stromal cells, the initial site of insult, as well as granulosa cells which exhibit a greater magnitude of DXR-induced DNA damage [23]. Oocytes do not exhibit significant DXR-induced DNA damage until 12 hrs post-injection. This comparatively late oocyte DNA damage occurs only as a sequel to significant late-stage apoptosis in follicular granulosa cells, suggesting DNA damage in oocytes may be subsequent to follicular demise. While DXR-induced apoptosis is apparent in growing follicles 12 hrs post-DXR treatment, apoptotic primordial follicles are not detected until 48 hrs post-DXR treatment [23]. The complex ovarian capitulation to DXR indicates that a successful ovoprotective agent must protect each ovarian cell- and follicle-type.

Though permeant to the cell plasma membrane, DXR binds the proteasome and is co-translocated across the nuclear membrane as a complex [24]. This transport step provides a potential mechanism to intercept nuclear DXR accumulation utilizing high affinity proteasome inhibitors to compete for DXR binding. Proteasome inhibitors were originally developed as anti-cancer agents and several are either approved for clinical use or currently in trials. The proteasome conducts over 90% of cellular protein turnover [25], and the active proteasome complex is translocated across the nuclear membrane to regulate nuclear protein turnover [26]. The proteasome does not function physiologically as a drug transporter, but does mediate DXR nuclear accumulation [24]. Active site inhibitors MG-132 and Bortezomib (Bort) directly compete with DXR for binding to the proteasome and prevent DXR nuclear accumulation in L1210 cells [27]. MG-132 also prevents DXR-induced DNA damage in cardiac-derived H9C2 cells [28]. Well-tolerated in normal tissue, Bort is clinically used as an anti-cancer agent to treat myelomas and lung cancers, and is currently being tested as an adjuvant for a variety of other cancers [6], [29], [30]. Bort-induced toxicity in cancer cells arises from their need for NF-κB turnover to facilitate DNA transcription and rapid cell division [6], [31]. Non- or slowly-dividing cells in normal tissue are better able to tolerate Bort action.

The present study utilized an adolescent mouse model to test the hypothesis that Bort pretreatment protects the pubertal ovary from acute DXR-induced injury and could thus serve such a role in humans. The CD1 strain is commonly used in toxicology studies, and was chosen because it is an outbred strain more likely to reflect heterogeneity in the human population as opposed to an inbred strain, and CD1 mice are robust breeders with large ovaries and litter sizes, allowing measurement of reduced litter size. Bort pretreatment prevented primary DXR-induced DNA damage in all ovarian cell types through 24 hrs post-injection, attenuated subsequent DXR-induced γH2AFX phosphorylation, and activation of downstream apoptotic pathways, specifically reducing the apoptotic response to DXR in preantral follicles. The mechanism of protection involves decreased DXR accumulation within ovaries, consistent with blockade of nuclear transport. Consistent with decreased follicular apoptosis, Bort improved the number of litters per mouse following DXR treatment, provided a recovery of litter size over time, and increased birth weight of the pups. This study demonstrates Bort pretreatment is a promising tool to reduce DXR-induced ovarian toxicity *in vivo*, and improve long-term fertility and fecundity, particularly for adolescents.

## Methods

### Chemicals

Bort was obtained from Sigma, complete protease inhibitors from Roche, DXR from the UW-Madison Chemo Pharmacy, and all other chemicals from Fisher.

### Lysate preparation and Western blots

Ovaries were homogenized, protein quantified, and WBs were conducted as previously described [10]. Blots were probed with rabbit anti-γH2AFX antibody (Abcam, 1∶500), and mouse anti-β actin (Sigma, 1:10,000). WBs were scanned and analyzed using the 5 LiCor Odyssey System (UW-Small Molecule Screening Facility) [10].

### Mice

This study was conducted in accordance with the Guide for the Care and Use of Laboratory Animals and the Animal Welfare Act. Procedures were approved by the Medical School Animal Care and Use Committee of the University of Wisconsin (UW)-Madison. Animals (Charles Rivers) were purchased through and housed in the UW Animal Care Facility in Inovive system cages, accredited by the Association for Assessment and Accreditation of Laboratory Animal Care, and provided with standard care as well as free access to food and water. Light cycle in the facility starts at 6 a.m., dark cycle at 6 p.m. with an ambient room temperature of 72°C. Four-week old female CD1 mice were treated with 0.143 mg/kg Bort or vehicle control, followed by 20 mg/kg DXR (twice the human equivalent dose) or saline via intraperitoneal injection (200 µL total volume/injection) 1 hr later. Three mice were injected per time point, 2, 4, 6, 12, and 24 hrs, as indicated. Experiments were performed in triplicate or quadruplicate for a total of 72 mice. To minimize variability in time from injection to tissue harvest (particularly for short time points), mice were injected and harvested in the same order according to treatment group: control, DXR, Bort+DXR, Bort. Sample size was based on statistical significance achieved in our previous publication describing *in vivo* DXR insult in the mouse [23]. Utilizing one ovary from each mouse for the comet assay and the contralateral ovary for protein extraction or TUNEL assay reduced the total number of mice needed for the study and allowed pairwise comparison of the data. Mice were housed with 3–4 animals per cage following injection. Injections were performed early in the morning, typically by 8:00 a.m. in the Biological Safety Hood within the UW Animal Care Facility to minimize mouse handling and transport. Mice were euthanized with CO_2_, and ovaries processed as previously described [23].

Breeding females had diets supplemented with breeder chow, irradiated seeds, and hydrating gel packs (Clear H_2_O), where males were maintained on regular chow when not with females (Harlan Maintenance Diet 8604; protein 24%, fat 4%, fiber 4.5%, and Harlan Irradiated Diet 2919; protein 19%, fat 9%, fiber 5%). The extra nutritional support provided by the hydrating food gel was to minimize weight loss caused by chemotherapy treatment. Mice were monitored daily for body score assessment including body weight and fur condition. DXR-treated mice exhibited increased rates of dystocia; mice presenting with dystocia were treated with saline injections and heat lamps, and monitored several times throughout the day of presentation. If mice failed to deliver within 24 hours of presenting with dystocia, they were euthanized per approved protocol. Four replicate breeding trials were conducted with 4–8 female CD1 mice per treatment group per trial for a total of 96 mice. Per above, four week-old adolescent females were injected with 0.143 mg/kg Bort or vehicle control, followed 1 hour later by 10 mg/kg DXR or saline via intraperitoneal injection (100 µL total volume/injection). To decrease susceptibility to infection following chemotherapy immune suppression, all breeding mice were treated for 2 weeks with Baytril (0.5 mL/300 mL ddH_2_O bottle) starting at 6 weeks of age. Females were mated to proven stud males at 8 weeks of age, and removed to separate cages for births when visibly and palpably pregnant. Each male was paired with no more than two females at a time. Females were returned to male cages within 24 hours of litter delivery. Males were rotated through the females to minimize any male-related infertility factors. One male was identified as infertile, and removed from the trial. Females not visibly pregnant remained with males continuously until they achieved pregnancy or until the end of the trial at 8 months of age. Pups were weighed on PND1 and euthanized per approved protocol. At the end of the breeding trial, surviving females were euthanized by cervical dislocation following a terminal blood draw (forearm) per approved protocol, and ovaries were removed and weighed (dry weight).

### Neutral Comet Assay

Ovaries were processed to provide enriched populations of granulosa cells/oocytes, and stromal/thecal cells for neutral comet assay [10], [23]. Briefly, follicles were punctured with a needle to release granulosa cells and oocytes from large follicles. The remaining tissue enriched for stromal and thecal cells was processed with 25% collagenase to obtain a single-cell suspension. Though not pure, this enriched population is referred to as “stromal/thecal cells” for simplicity. Oocytes were easily distinguished on the comet slides as they are significantly larger than granulosa cells. At least 100 granulosa and stromal/thecal cells and 50 oocytes were imaged from blinded slides per time point per mouse (3 mice/replicate) [10], [23]. The comet moment to quantify DNA damage was scored using CometScore software. Data were normalized to control for each experiment and allow pooling across experiments.

### Fluorescence Microscopy

Images were collected using a Nikon A1 laser scanning microscope with a motorized stage to image the entire section at 400X magnification. Each spectral image was taken at the Z plane providing maximal signal in the section utilizing identical laser settings with the spectral scan head, exciting at 488 nm and collecting emissions from 520 nm through 720 nm at 10 nm intervals [23]. Images presented were overlays of all emissions, thereby including all signal over the DXR emission range. Total DXR fluorescence was measured in each section image using Nikon Elements, quantifying fluorescent intensity at each emission wavelength.

### TUNEL staining

Apoptosis was detected utilizing ApopTag Plus Fluorescein In Situ Apoptosis Detection Kit, as previously described [23], using the contralateral ovary from mice used for the comet assay at 12 hrs post-DXR. Nuclei were counterstained with 0.5 µg/mL Propidium Iodide. Apoptotic index was determined only including follicles containing a visible oocyte to allow positive morphological analysis; follicle types were differentiated by standard morphology and size ranges. Primary, secondary, and antral follicles were considered positive if they had ≥4 TUNEL-positive granulosa cells [32].

### Statistics

Graphs and ANOVA analyses were generated using OriginLab. All ANOVAs were conducted including means comparisons as indicated, set at p≤0.05.

## Results

### Bortezomib pretreatment prevents DXR-induced DNA damage

**Figure 1:**
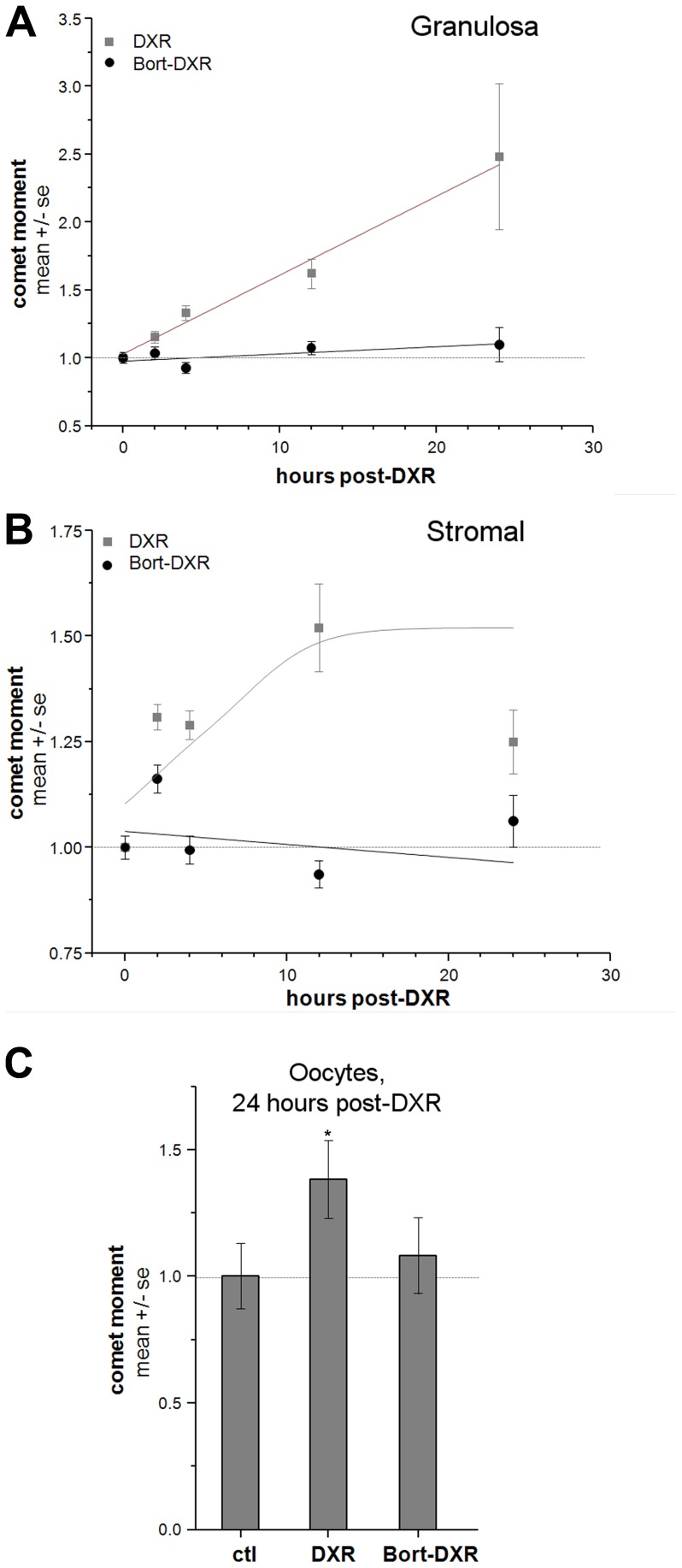
Bort pretreatment prevents DXR-induced dsDNA breaks in ovarian cells. Summary data quantify dsDNA damage as the comet moment utilizing the comet assay. Trend lines included for visualization. Panels summarize DNA damage in granulosa cells (*A*.) and stromal cells (*B*.) as time post-DXR injection plotted against comet moment. *C*. Bar graph summarizes DNA damage in oocytes 24 hrs post-DXR injection. n = 3 animals/group/time point/replicate, 4 replicates total. *p<0.05, one-way ANOVA.

We tested the hypothesis that Bort pretreatment prevents DXR-induced DNA damage utilizing the neutral comet assay to quantify dsDNA damage in all ovarian cell types over a 24-hr post-injection period. Four week-old adolescent mice were pretreated with 0.143 mg/kg Bort or vehicle control via i.p. injection 1 hr prior to 20 mg/kg DXR i.p. injection. The Bort dose corresponds to 1/3^rd^ the lowest human equivalent dose utilized in chemo regimens (0.43 mg/m^2^ vs. 1.3 mg/m^2^), while the DXR dose is double the standard human equivalent dose, permitting direct imaging of DXR fluorescence. Quantifying dsDNA breaks as the comet moment in individual cells revealed that our low dose Bort treatment prevented time-dependent DXR-induced DNA damage in granulosa and stromal/thecal cell-enriched populations in ovaries from treated mice 2–24 hrs post-injection (Fig. 1a and 1b, respectively). While DXR induced a linear increase in granulosa cell comet moment to 250% of control values, Bort-DXR-treated mice maintained comet moment within 10% of control values (Fig. 1a). Similarly, DXR increased comet moment in stromal cells to 150% of control values, whereas Bort pretreatment maintained DNA damage within 16% of control (Fig. 1b). Not only was the initial onset of DNA damage blocked, but there was no delayed DNA damage response in the Bort-pretreated animals. DXR is rapidly cleared from the systemic circulation (within 15–30 minutes), and the lack of DNA damage over a 24-hr period suggests Bort protection was maintained throughout the DXR clearance timeframe. These data indicate that Bort pretreatment provides protection to the ovary across the entire acute DXR insult period rather than simply delaying DNA insult. A lack of increase in oocyte comet moment (Fig. 1c) demonstrated Bort also completely blocked DXR-induced DNA damage in germ cells at 24 hrs post-injection. Oocytes were not examined for DNA damage at earlier time points, as we previously demonstrated absence of DXR-induced DNA damage until at least 10 hrs post-injection. The comet assay therefore revealed Bort completely prevented DXR-induced DNA damage in all ovarian cell types during the first 24 hrs.

### Bortezomib prevents H2AX phosphorylation in response to DXR

**Figure 2:**
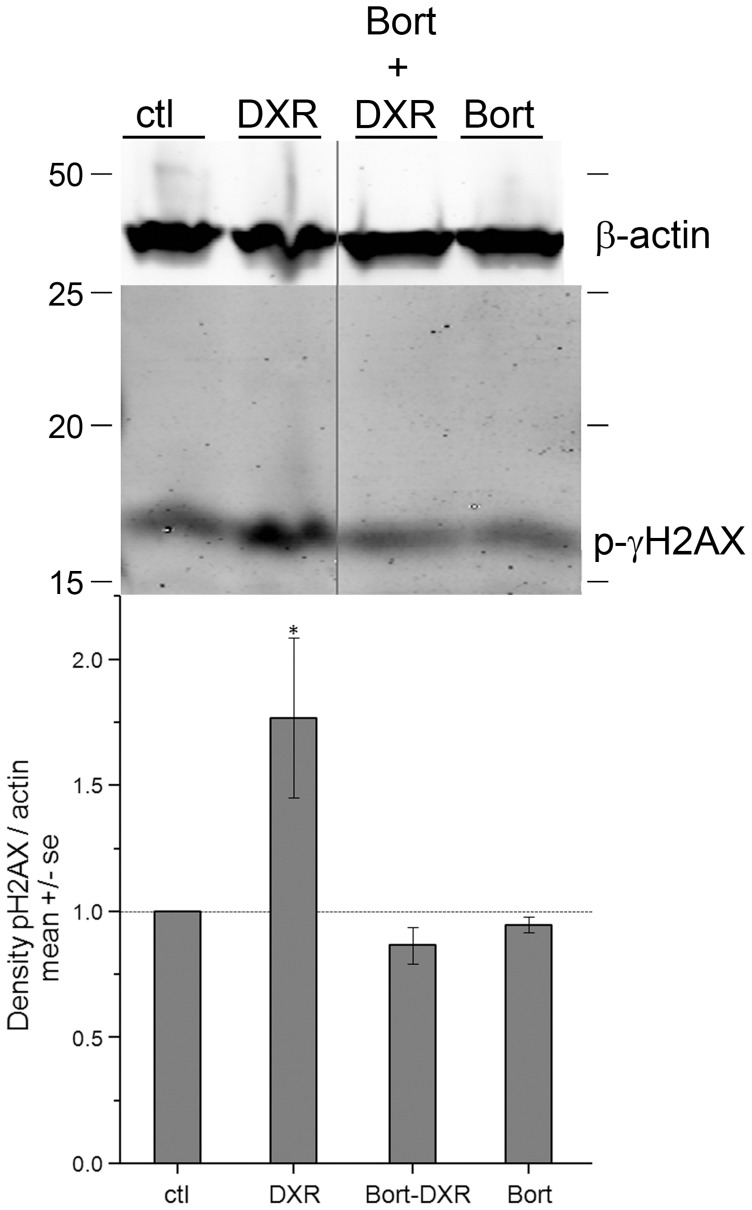
Western blot with corresponding quantification reveals DXR-induced changes in H2AFX phosphorylation were blocked by Bort pre-treatment. Blot probed with anti-phospho H2AFX antibodies revealed an increase in density of the corresponding 17 kDa band in ovarian lysates harvested 6 hrs post-DXR injection compared to ctl, which is absent in Bort-pretreated samples. *p<0.05, one-way ANOVA, Tukey means comparison. Blot shows β-actin as the loading control. N = 3 blots/quantification.

To determine whether Bort prevents the cellular response to DXR-induced DNA damage, Western blots (WBs) of ovarian lysates were probed for phosphorylated γH2AFX. Confirming the lack of DNA damage quantified in the comet assay, Bort pretreatment attenuated DXR-induced γH2AFX phosphorylation (activation), the earliest cellular response to dsDNA breaks. DXR increased γH2AFX phosphorylation, demonstrated by increased intensity of the corresponding 17 kDa band on WBs of ovarian lysates harvested 6 hrs post-injection and probed with anti-phospho-γH2AFX antibodies (Fig. 2a). The phospho-γH2AFX response was lacking in Bort-DXR-treated mice, demonstrating effective shielding. Bort-treated mice did not exhibit increased phospho-γH2AFX, demonstrating Bort alone does not activate γH2AFX. Bort pretreatment blocked γH2AFX phosphorylation in response to DXR, consistent with the prevention of DNA damage demonstrated in Fig. 1.

### Bortezomib shields follicles from DXR-induced apoptosis

**Figure 3:**
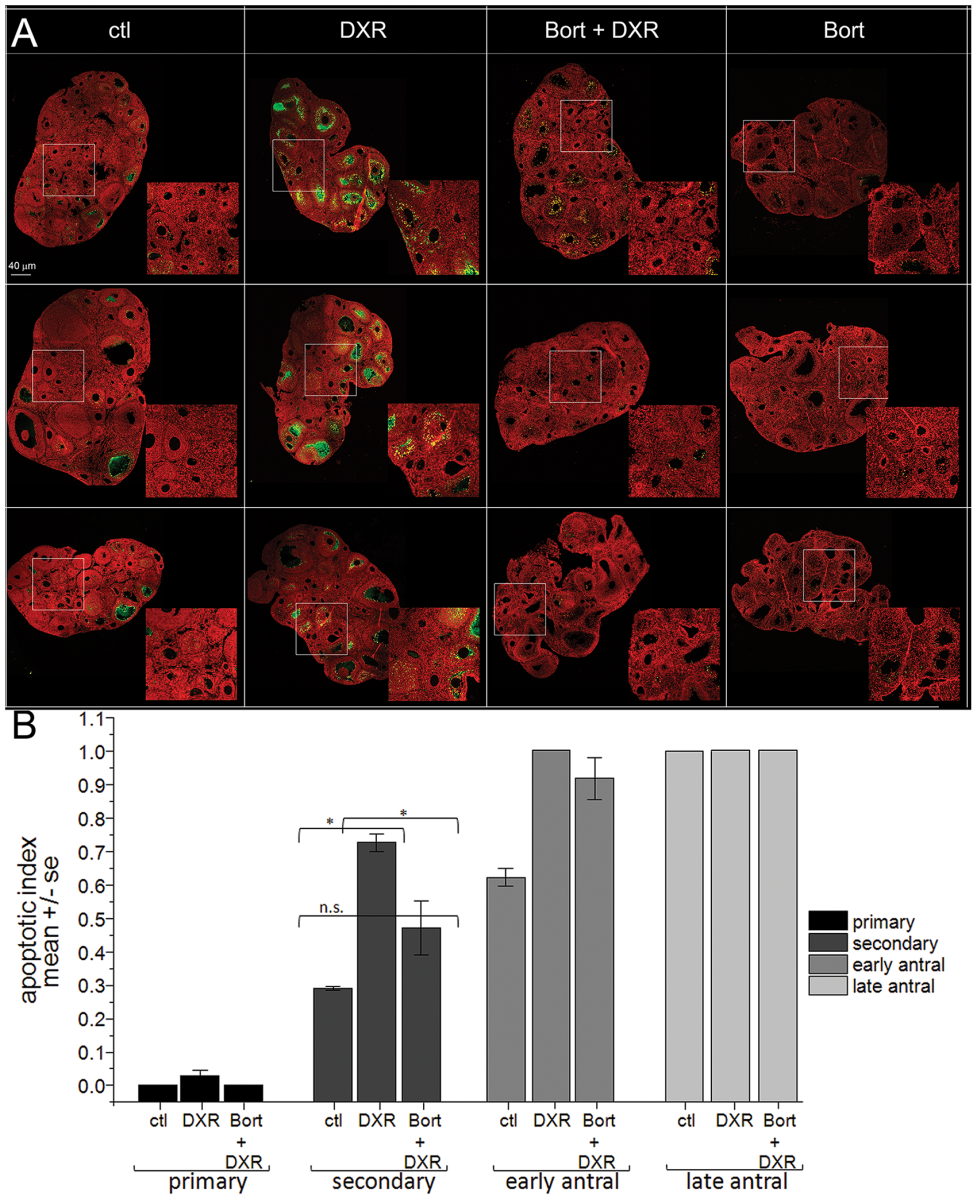
Bort pretreatment prevented DXR-induced apoptosis in mouse ovarian follicles. *A*. Micrographs of mouse ovaries stained with TUNEL (green) or PI (red, nuclei), bar  = 40 µm). Representative images from 3 different mice are shown for each treatment condition. Insets are digital magnification. *B*. Bar graph quantifies the apoptotic index per follicle class calculated as fraction apoptotic/total follicles for each class. n = 4 mice *p<0.05, one-way ANOVA, Bonferroni means comparison. The very low levels of TUNEL-positive follicles in Bort-treated mice were not quantified.

To ascertain whether Bort pretreatment prevents DXR-induced follicular apoptosis, ovaries were harvested from mice treated with control vehicle, Bort, DXR, or Bort + DXR, 12 hrs after chemo administration (Fig 3A, micrographs). Under stringent scoring criteria labeling an entire follicle as apoptotic if it contained ≥4 TUNEL-positive granulosa cells [32], Bort pretreatment reduced the DXR-induced doubling of apoptotic-positive secondary follicles to levels not different from control (Fig. 3b). Primordial follicles were not assessed as they do not exhibit quantifiable levels of apoptosis until 48 hours post-DXR injection [23]. Bort also decreased apoptosis in primary follicles indicating Bort attenuated DXR-induced follicular demise *in vivo*, preserving the growing, preantral follicles key for subsequent fertility.

### Bortezomib pretreatment reduces total DXR accumulation in the ovary

**Figure 4:**
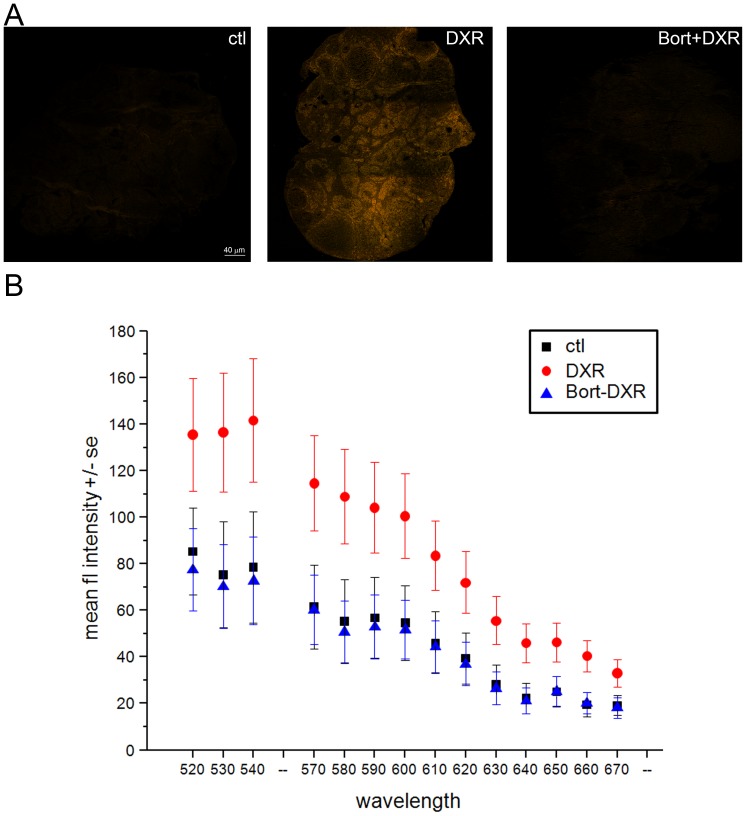
Bort pretreatment prevented DXR accumulation in the mouse ovary. *A*. Micrographs of mouse ovarian sections obtained by spectral confocal imaging (ex. 488 nm, em. 520–720 nm). Images are overlays of all collected emission wavelengths. Bar = 40 µm. To ensure control autofluorescence was visible in print, images were adjusted equally to a threshold of 140 in Photoshop with no other image enhancements. *B*. Graph plots mean fluorescence intensity +/− SEM quantified from raw DXR fluorescence in ovarian sections representing the top, middle, and bottom third of the ovary. Emission profiles at 550–560 nm (cold finger) were not collected by the microscope to prevent direct detection of the excitation laser at that wavelength. Confocal parameters were identical from one sample to the next. DXR points are statistically significant from control and Bort-DXR with p<0.05, one-way ANOVA, Bonferroni means comparison.

Previous studies by Kiyomona et al. [27]demonstrate that DXR directly binds the proteasome; both binding and DXR nuclear transport and accumulation *in vitro* are blocked by proteasome inhibitors. To test the hypothesis that Bort prevents DXR-induced ovarian DNA damage by reducing DXR accumulation in the ovaries *in vivo*, we quantified ovarian DXR utilizing the drug's autofluorescence [23]. Spectral images of ovarian sections (Fig. 4a, spectral emission composites) demonstrated Bort pretreatment decreased DXR fluorescence to baseline levels (Fig. 4b, quantification). These data are consistent with the proposed mechanism in which Bort prevents DXR entry to cell nuclei by competing with DXR for proteasome binding. While further microscopy studies at higher magnification will facilitate single cell level analysis to distinguish nuclear from cytosolic DXR accumulation, our data demonstrate a Bort-mediated lack of DXR fluorescence across all ovarian follicle types and stromal tissue.

### Mice pretreated with Bortezomib recovered litter size over time compared with DXR-only treated mice

**Figure 5:**
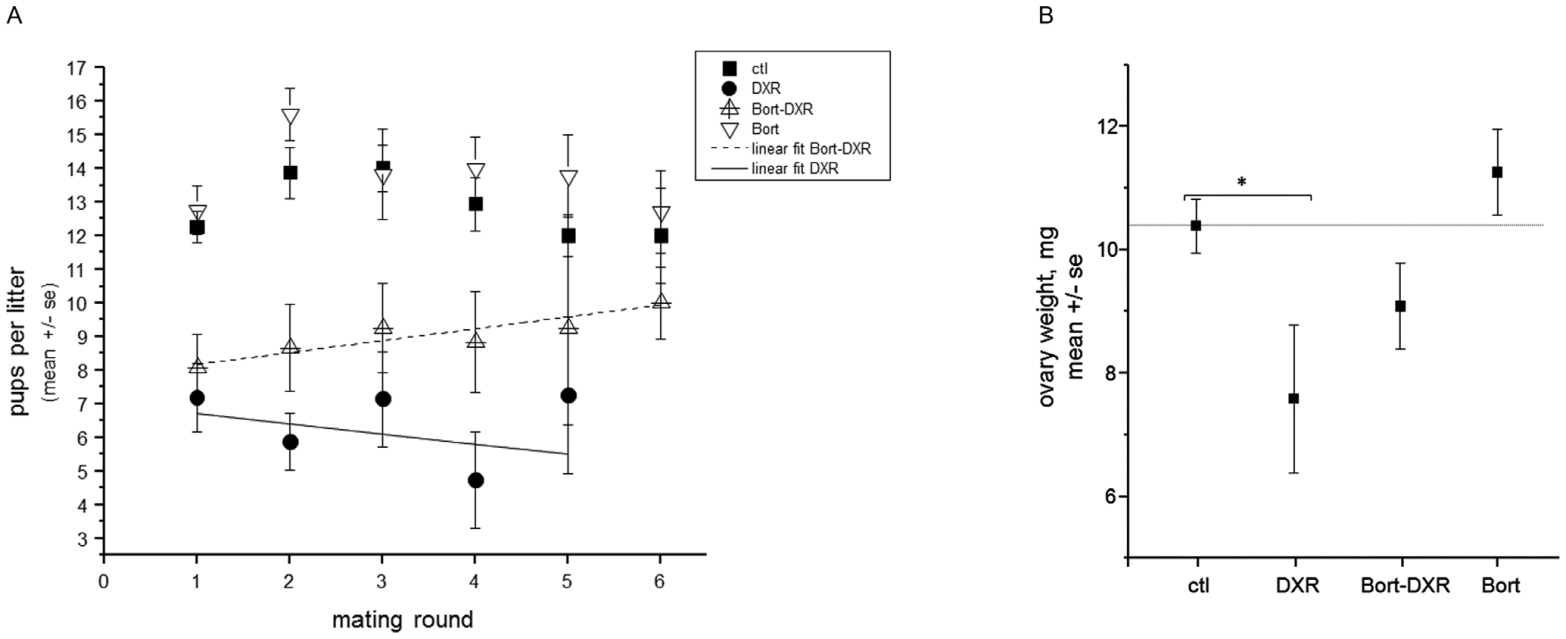
Bort pretreatment increases pup number per litter and ovarian weight following DXR. Number of pups birthed in each litter were counted and weighed for each treatment group. *A*. Plot depicts the mean number of pups per litter (± SE) for each litter number for each dam treatment group. Linear fit for Bort-DXR has a slope of 0.35±0.06 SE, DXR linear slope  = −0.31±0.04. *p = 0.033 for DXR vs. Bort-DXR, two-way ANOVA. Only 1 DXR-treated mouse achieved a sixth litter; the point is not plotted as no error can be calculated. *B*. Graph plots mean weight of ovary (mg) ± SE for each treatment group at 8 months of age [ctl = 10.4±0.4 mg (n = 36), DXR = 7.6±1.2 mg (n = 8), Bort-DXR = 9.1±0.7 mg (n = 22), Bort = 11.3±0.7 mg (n = 32)]. DXR induced a significant reduction in ovary weight (p<0.05, one-way ANOVA). Bort-DXR and Bort treatment groups were not significantly different from control.

Breeding trials were conducted to determine whether Bort pretreatment also increased female fertility and fecundity following DXR. Female mice were treated with chemotherapy +/− Bort pretreatment at 4 weeks of age per the acute experiments utilizing a reduced DXR dose of 10 mg/kg to limit cardiotoxicity and thereby increase animal viability. Females were mated at 8 weeks of age, and litters assessed through 6 consecutive breeding rounds (see Methods). Figure 5 demonstrates that while both DXR and Bort-DXR mice exhibited an initial decrease in litter size from 12.2±0.5 (ctl) to 7.2±1.0 and 8.1±1.0 pups, respectively, Bort-DXR-treated mice progressively recovered litter size over time to 9.3±2.9 and 10.0±1.1 SE pups by litters 5 and 6, respectively (∼7 months post-DXR, linear slope  = 0.35±0.06). DXR-treated mice, in contrast, maintained a reduced litter size across all mating rounds (7.3±2.3 pups at litter 5), with a linear slope of −0.31±0.40. Two-way ANOVA with a Bonferroni means comparison confirmed a difference in litter size between groups (p = 0.04 for DXR vs. Bort-DXR through 5 litters), but not across birth number (litter round). While 38% of Bort-DXR mice (4 of 11 surviving) achieved 6 litters by 7 months post-injection, only 1 of 4 (surviving) DXR-treated mice achieved a sixth litter; the latter point is not included in Fig. 5 as no error can be calculated. Bort treatment alone was well tolerated, with litter sizes not significantly different from control-treated animals (Fig. 5). Taken as an average across all litters, DXR reduced litter size to 6.8±0.6 from a mean of 13.0±0.3 for control-treated animals. Bort-DXR treated mice had an overall average litter size of 8.8±0.6, improving the litter size over DXR-treated mice. Mice treated with Bort alone had an average litter size of 13.8±0.4. These data illustrate that while the Bort dose tested did not completely recover litter size to control values, pretreatment with the proteasome inhibitor increased litter size in DXR-treated animals across 5 mating rounds, demonstrating long-term fecundity improvements. Concomitant with improved litter size, Bort-DXR treated mice had ovaries with a larger mass than DXR treated mice at the end of the breeding trial (8 months of age, Figure 5b). DXR reduced ovarian weight to 7.6±1.2 SE mg, in contrast to 10.4±0.4 mg for control-treated animals (p<0.05, one-way ANOVA). Bort-DXR ovaries were not smaller in mass than control ovaries, however, at 9.1±0.7 mg, demonstrating an improvement in retained ovarian mass following DXR. Consistent with a large litter size, Bort-treated animals had ovaries that averaged 11.3±0.7 mg at 8 months of age, and were not different from control. Taken together, these data demonstrate that Bort pretreatment provides long-term protection to the ovary following DXR treatment, and improves litter size over time and maintains ovarian mass.

### Bortezomib pretreatment improved pup weight following DXR

**Figure 6:**
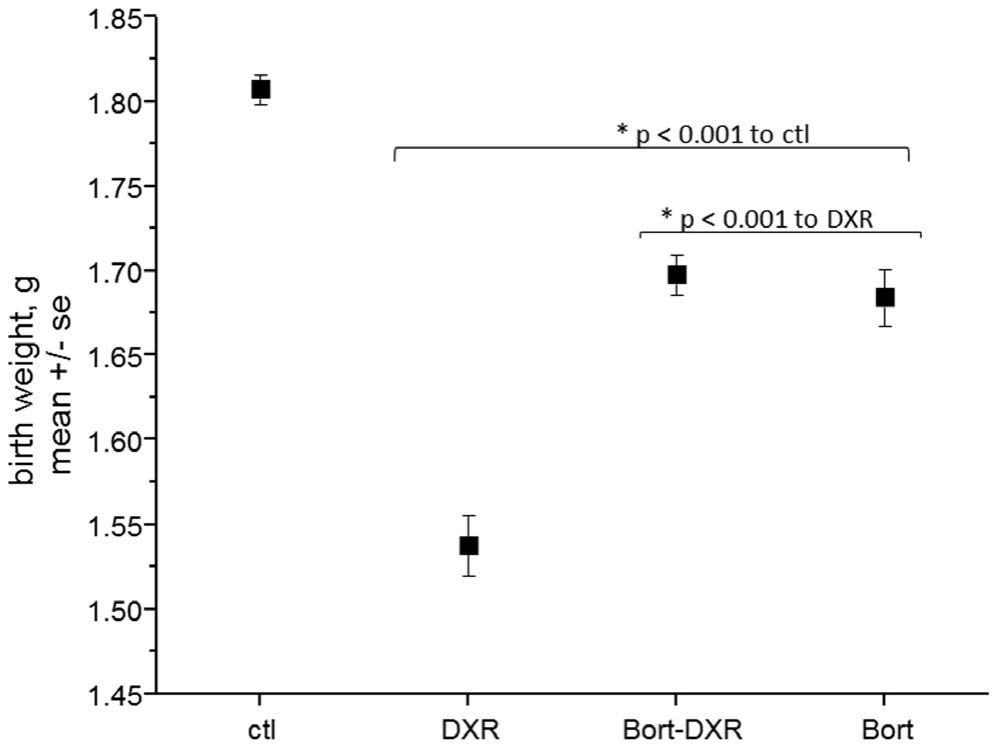
Bort pretreatment improves pup weight following DXR. Pup weight, g, is plotted ± SE for each treatment group. DXR reduced pup birth weight to 86% of control, where Bort treatment groups improved pup weight to 94% of control. Asterisks denote p<0.001, one-way ANOVA with Bonferroni means comparison (n = ≥170 pups per treatment).

While it is widely held that infants born post-chemotherapy do not exhibit ramifications of the chemotherapy treatment women received prior to conception, we found that DXR treatment decreased pup birth weight from 1.8 g±0.01 (ctl) to 1.5 g±0.02 (Fig. 6, p<0.001 one-way ANOVA). Bort-DXR treated mice had pups with an average birth weight that while still lower than control (p<0.001), was an increase over DXR-treatment alone, at 1.7 g±0.01 (p<0.001). The DXR birth weight was reduced by 16% compared to control pups, whereas Bort-DXR pups exhibited only a 6% reduction in birth weight. Pups born to females treated with Bort alone had a birth weight not different from Bort-DXR at 1.7 g±0.02, but both groups exposed to Bort still exhibited a 6% decrease from ctl (p<0.001). There were no obvious anatomical defects in pups from any of the treatment groups upon delivery. The change in pup birth weight was not due to uterine crowding, as control animals exhibited large litter sizes, but also large pups. In contrast, DXR-treated animals exhibited the smallest litter sizes, and the smallest pups. Indeed the birth weight∶litter size ratio for DXR-treated animals was >0.2, whereas the average ratios for control and Bort-DXR treated animals were identical at 0.13. These data demonstrate a surprising DXR-induced reduction in pup birth weight independent of litter size that is improved by pretreatment with Bort.

### Bortezomib pretreated mice exhibited a longer fertility window than mice treated with DXR alone

**Figure 7:**
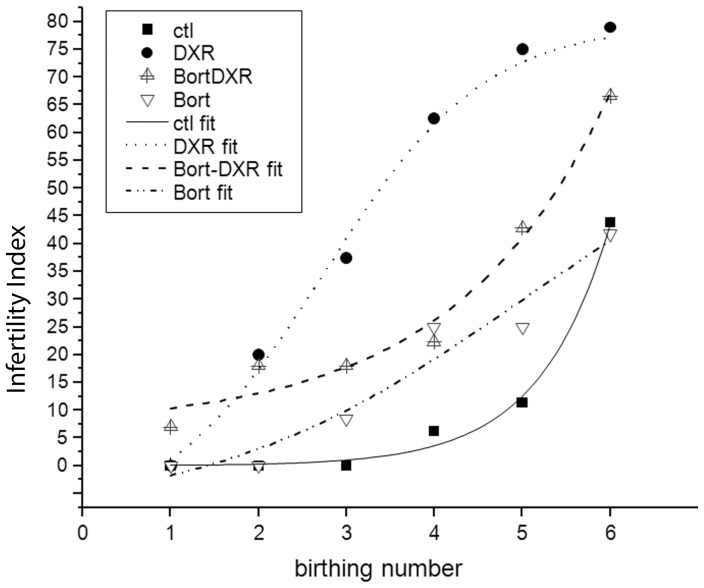
Bort pretreatment improves the infertility index following DXR treatment. Plot represents the ‘infertility index’ plotted as the percentage of surviving animals from each treatment group that fail to achieve the next birth as a function of birth (litter) number. Symbols correspond to control, DXR, Bort –DXR and Bort treatment groups as indicated. DXR points in the linear range were statistically different from Bort-DXR, Bort, and control (p<0.05, one-way ANOVA).

DXR-treated mice exhibited a significant loss in fertility over time, with over 50% of surviving animals failing to achieve more than 3 litters (Fig. 7). The “infertility index” is plotted vs. birth number (delivery), where the infertility index is defined as the percentage of surviving females that fail to deliver after the subsequent mating round. While exhibiting reduced fertility compared to control animals, Bort-DXR treated animals demonstrated significantly improved fertility compared to DXR-treated mice (p<0.05, two-way ANOVA over the linear range). The infertility index of Bort-DXR treated animals did not rise above 50% until after 5 litters, at which point both control-treated and Bort-treated animals started to exhibit an age-related reduction in delivery rates. Bort-treated animals were not different from control (p>0.05, two-way ANOVA over the linear range). Bort alone was well-tolerated by the mice, with 90% survivorship by the end of the trial (Figure S1). DXR treatment caused an initial decrease in survivorship of exposed animals to 75%, followed by a continuous decline to 25% survivorship by the end of the trial (Figure S1). While some of the DXR-treated mice declined due to weight loss despite nutritional support, much of the morbidity was due to dystocia despite intervention. Bort-DXR treated mice declined more slowly than DXR, but Bort pretreatment did not improve long-term survivorship (Figure S1), with morbidity attributed to dystocia. These data demonstrate the Bort pretreatment dose used in this study expands the reproductive window for DXR-treated mice, allowing more litters per mouse and increased litter size.

## Discussion

This study presents the clinically-approved anti-cancer proteasome inhibitor, Bort, as a promising candidate for *in vivo* prophylactic prevention of anthracycline toxicity in the normal ovary. The adolescent mice utilized in this study model a human patient population for whom current oncofertility treatments are limited. Pharma shields like Bort, however, that prevent key markers of chemo-induced damage when the prophylactic is given immediately prior to each chemo dose, should be effective in pre-menopausal patients of any age regardless of cancer type. This is particularly important for patients who lack local access to IVF specialists, simply cannot afford current treatment alternatives, or have personal objections to embryo and oocyte cryopreservation. Drug-based ovarian shields can be administered by oncologists as part of the chemotherapy protocol, and thus minimize the need for local fertility specialists. Such prophylactics thus have the potential to improve current oncofertility practices and compliment cryopreservation-based fertility technologies.

While Bort blocked DXR-induced dsDNA breaks in all ovarian cell types, including oocytes, over the entire 24-hr period of the present study, there was a small rise in comet moment in stromal cells from Bort-DXR mice at 2 hrs, followed by a drop back to baseline. Whether this represents transient DNA damage that is repaired, or a population of stromal cells that sustain damage, undergo necrosis, and hence are not detected at later time points can be assessed in future studies. In addition, one of three replicates of stromal cells isolated from DXR-treated mice at 24 hrs showed an apparent decrease in comet moment, resulting in a decreased mean. This was not observed in the larger number of mice used in our initial characterization of acute DXR insult to the mouse ovary [23]. As previous work has demonstrated significant necrotic deterioration of stromal tissue by 12–24 hrs post-DXR, it is most likely the apparent decrease in damage is an artifact due to loss of affected cells.

Adding to other fertoprotective agents under development, including FTY720, dexrazoxane, AS101, sphingosine-1-phosphate, imatinib, tamoxifen and goserelin [7]–[13], proteasome inhibitors represent promising ovoprotective agents as the drugs were developed as anti-cancer agents and enhance, rather than interfere with, anti-tumor efficacy of traditional chemo agents [6]. In addition, future development of ovarian-targeting mechanisms may allow specific delivery of ovoprotective drugs. Below the lowest human equivalent dose used in chemo regimens (human equivalent dose 1.3 mg/m^2^), the Bort dose tested here (0.43 mg/m^2^) should be well-tolerated in patients. The ability to kill cancer cells while protecting normal tissue cells seems contradictory, but is based on the same therapeutic window concept that allows chemo to kill cancer without destroying the patient from whom the original cancer arose. Slower-dividing (normal) cells are less susceptible to chemotherapy agents which target cellular requirements for rapid division. New generation proteasome inhibitors that increase anti-cancer potency and decrease systemic side effects may provide further improvements over Bort as ovoprotective agents.

Cancer patients often receive chemotherapy as a combination of drug classes and in conjunction with radiation therapy. Effectively shielding the ovary from such a multipronged cancer treatment may indeed require a multipronged shield. Future studies will be needed to determine whether Bort is effective in shielding the ovary from other chemotherapy classes and whether preventing DXR toxicity to the ovary still improves fertility and endocrine function in patients receiving combination chemotherapy including highly ovotoxic drugs like cyclophosphamide. In addition, trials combining Bort with drugs like FTY720 [7] to prevent radiation damage to the ovary and AS101 or tamoxifen [9], [12] to prevent cyclophosphamide toxicity to the ovary will be needed to determine whether a combined ovoprotective agents can provide an effective shield without compromising cancer therapy.

We previously demonstrated Dexrazoxane (Dexra), a catalytic topoisomerase II (topoII) inhibitor and antioxidant, can shield the mouse ovary from DXR *in vitro* [10]. While both Dexra and Bort prevent DXR-induced double strand DNA breaks, the mechanistic models are different. As a catalytic topoII inihibitor, Dexra prevents the double strand DNA breaks that topoII creates as a normal part of DNA replication, presumably allowing slowly-dividing cells time to clear DXR. In addition, Dexra acts as an antioxidant and mitigates DXR-induced mitochondrial stress in the heart [33]–[38]. In contrast, Bort competes with DXR binding to the proteasome and therefore nuclear translocation [24], [27], thereby preventing DXR intercalation into the DNA. While side-by-side breeding trials would be needed to draw direct comparisons between the drugs, the advantage of Bort is significantly decreased risk for DNA mutations because DXR intercalation is blocked. Dexra has the advantage of potentially protecting from oxidative stress as well as DNA damage, but there are questions as to whether Dexra increases risk for secondary cancers. In contrast, Bort is currently used as an adjuvant to treat lung cancers and therefore predicted to enhance anti-cancer efficacy.

Long-term fertility and fecundity assessments demonstrated that Bort pretreatment improved reproductive health of dams and birth weight of pups following DXR treatment. While there was an initial drop in litter size, Bort-DXR treated animals exhibited a continuous rise in litter size over time. With an average of 28 days between litters, the gradual increase in litter size is consistent with maturation of a protected primordial follicle pool. Mouse primordial follicles take an average of 47 days to reach the antral stage, depending on how growth is measured [39], [40], [41], with only 3–6 primordial follicles activated daily [42]. As there was a similar duration of 28 days between chemotherapy injection and first pairing, there was insufficient time for the antral pool to be completely replaced by primordial follicles. Progressive recovery of fecundity over time is thus consistent with repopulation of a mature ovarian antral follicular pool with follicles recruited from the primordial pool during the post-DXR recovery period. The increase in the number of total litters each mouse delivered for Bort-DXR treated mice as compared to DXR alone was also consistent with protection of the primordial follicle pool, and a prolonged fertile period following DXR treatment. Future studies will determine whether Bort pretreatment improves follicle counts, and AMH and estradiol levels in the mice following DXR treatment, as well as providing an opportunity to define a minimal effective dose that maximizes the protective effects of Bort. Additional research will also determine the efficacy of pairing Bort pretreatment with each DXR dose in a traditional, multi-dose chemo protocol.

It is widely held that female cancer survivors who received only chemotherapy (not radiation), and are successful in achieving pregnancy, give birth to babies without complications [43]. Our data, however, demonstrated decreased pup birth weight following DXR. While the low birth weight did not appear due to uterine crowding based on calculations of birth weight/litter size, the DXR-only mice generally had lower body weight than controls, and low birth weight could be due to a decline in general health despite nutritional support. Bort-DXR treated mice had similar survival curves, but improved pup birth weight, however, suggesting that DXR-specific effects account for low pup birth weight and are improved by Bort protection. DXR-induced low birth weight observed in mice is consistent with previous reports that survivors of childhood cancer are indeed at risk for giving birth to low birth weight babies. Data from the Childhood Cancer Survivor Study demonstrated that pediatric and adolescent female cancer patients treated with non-alkylating chemotherapy agents like DXR were more likely to deliver low-birth weight babies (<2500 g) compared with female siblings [43]. Low birth weight increases risk for developing high blood pressure, diabetes, and heart disease later in life. When corrected for other risk factors including smoking, females receiving DXR had the highest risk ratio of any chemotherapy agent at 1.92 (95% CI), very similar to the 2.15 risk ratio for low birth weight associated with radiation treatment, a well-accepted risk factor. In contrast, babies born from individuals receiving alkylating chemotherapy agents, typically deemed more ovotoxic, were not at increased risk for low birth weight (RR 1.19). Bar et al 2003 similarly demonstrated that adolescent patients treated with chemotherapy regimens that included DXR (median age 12 years) had low birth weights in subsequent pregnancies as compared to control patients, though offspring had no congenital malformations [44]. Our mice similarly lacked gross malformations at birth. Our study demonstrates that mice pre-treated with Bort prior to DXR had a significant improvement in pup birth weight, suggesting Bort may serve not only to improve fertility, but may also improve the health of offspring born to female cancer patients who receive DXR as part of their chemotherapy.

In conclusion, this study presents Bort as a promising ovoprotective agent delivered prior to DXR treatment and provides a model from which to develop additional drug-based approaches to preserve female reproductive health by *preventing* acute chemo insult. Drug-based chemoprotection may require a multipronged approach, but has the potential to overcome current obstacles in oncofertility by preserving ovarian endocrine function for patients receiving chemotherapy during childhood and adolescent years. Cost-effective and easily administered in a non-invasive manner, such ovoprotection may prevent long-term health complications currently associated with chemo-induced premature menopause.

## Supporting Information

Figure S1 **Survival Curves.** Percent survival is plotted as a function of time for each treatment group. Symbols correspond to control, DXR, Bort-DXR, and Bort treatment groups as indicated.
(TIF)
