# Supplementary figures and images for: Bortezomib Prevents Acute Doxorubicin Ovarian Insult and Follicle Demise, Improving the Fertility Window and Pup Birth Weight in Mice
Source: PLoS One. 2014 Sep 24;9(9):e108174. doi: 10.1371/journal.pone.0108174 (PMC4176970; doi:10.1371/journal.pone.0108174)

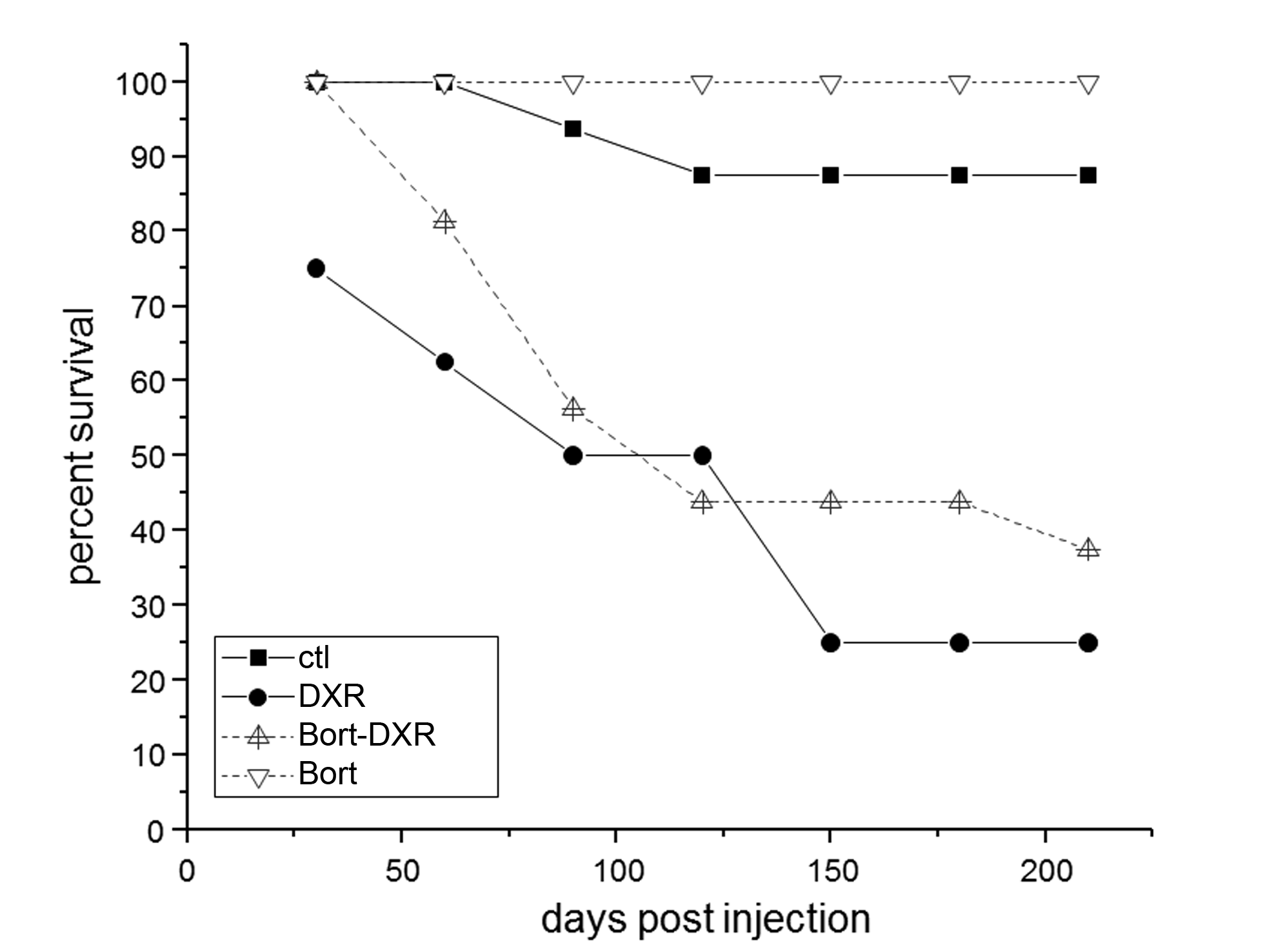

Supplement: Figure S1 — Survival Curves. Percent survival is plotted as a function of time for each treatment group. Symbols correspond to control, DXR, Bort-DXR, and Bort treatment groups as indicated. (TIF) [file pone.0108174.s001.tif]
